# Supplementary material for: Fibrotic microtissue array to predict anti-fibrosis drug efficacy
Source: Nat Commun. 2018 May 25;9:2066. doi: 10.1038/s41467-018-04336-z (PMC5970268; doi:10.1038/s41467-018-04336-z)
Supplement: Supplementary file 1 — Supplementary Information [file 41467_2018_4336_MOESM1_ESM.pdf]

Supplementary Information

Fibrotic lung microtissue array to predict anti-fibrosis drug efficacy

by Asmani M. et al.

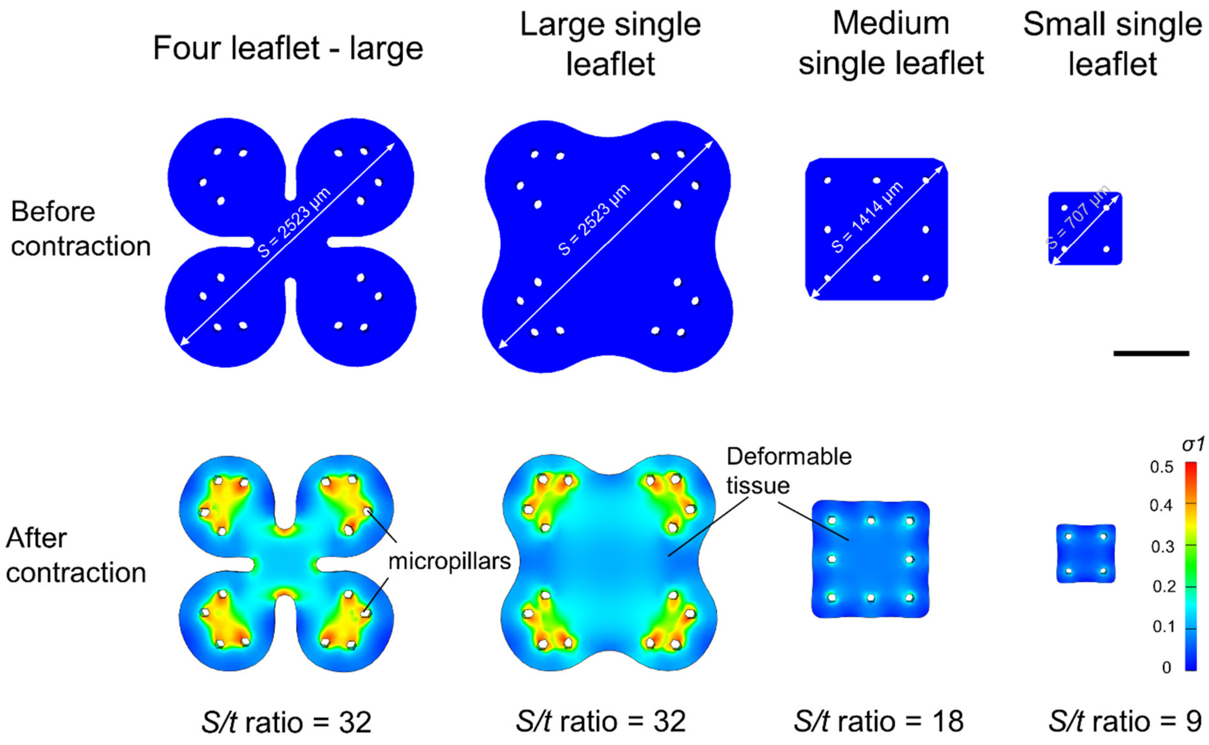

Supplementary Figure 1. Finite element (FE) models were used to study the effects of microtissue geometry and size on the evolution of contractile stress during microtissue formation. Top row, microtissue geometry before active contraction; bottom row, 1st principal stress contour was plotted on deformed microtissue geometry. Scale bar is 500 µm.

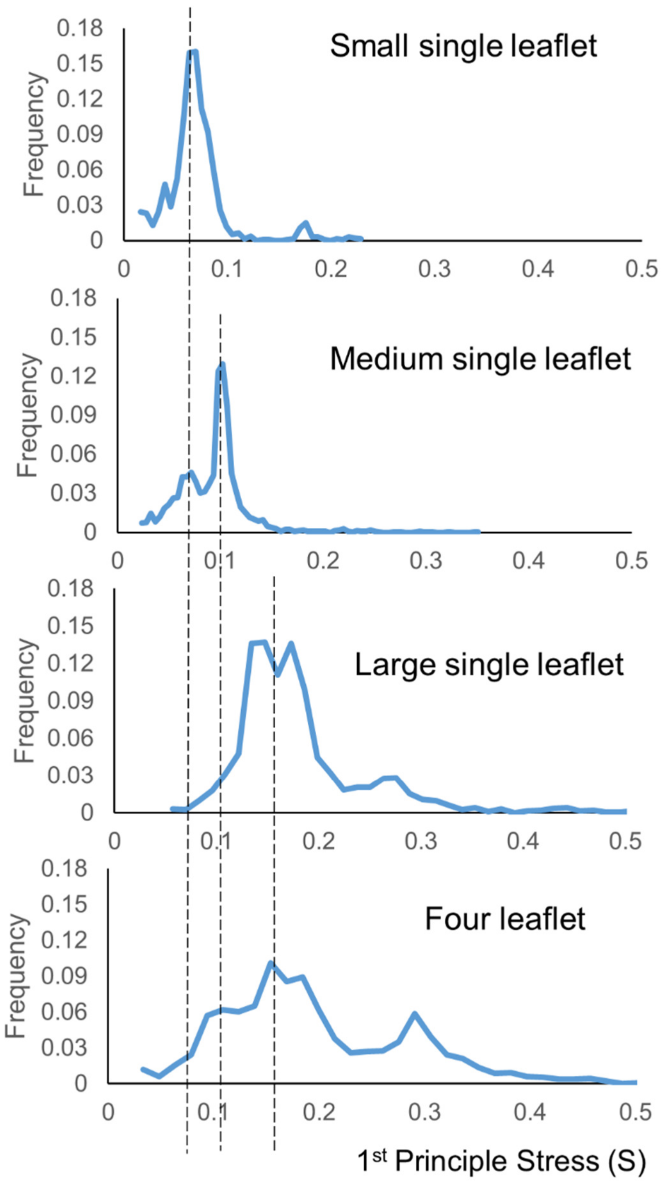

Supplementary Figure 2. Histograms of the 1st principal stress of four different microtissue designs.

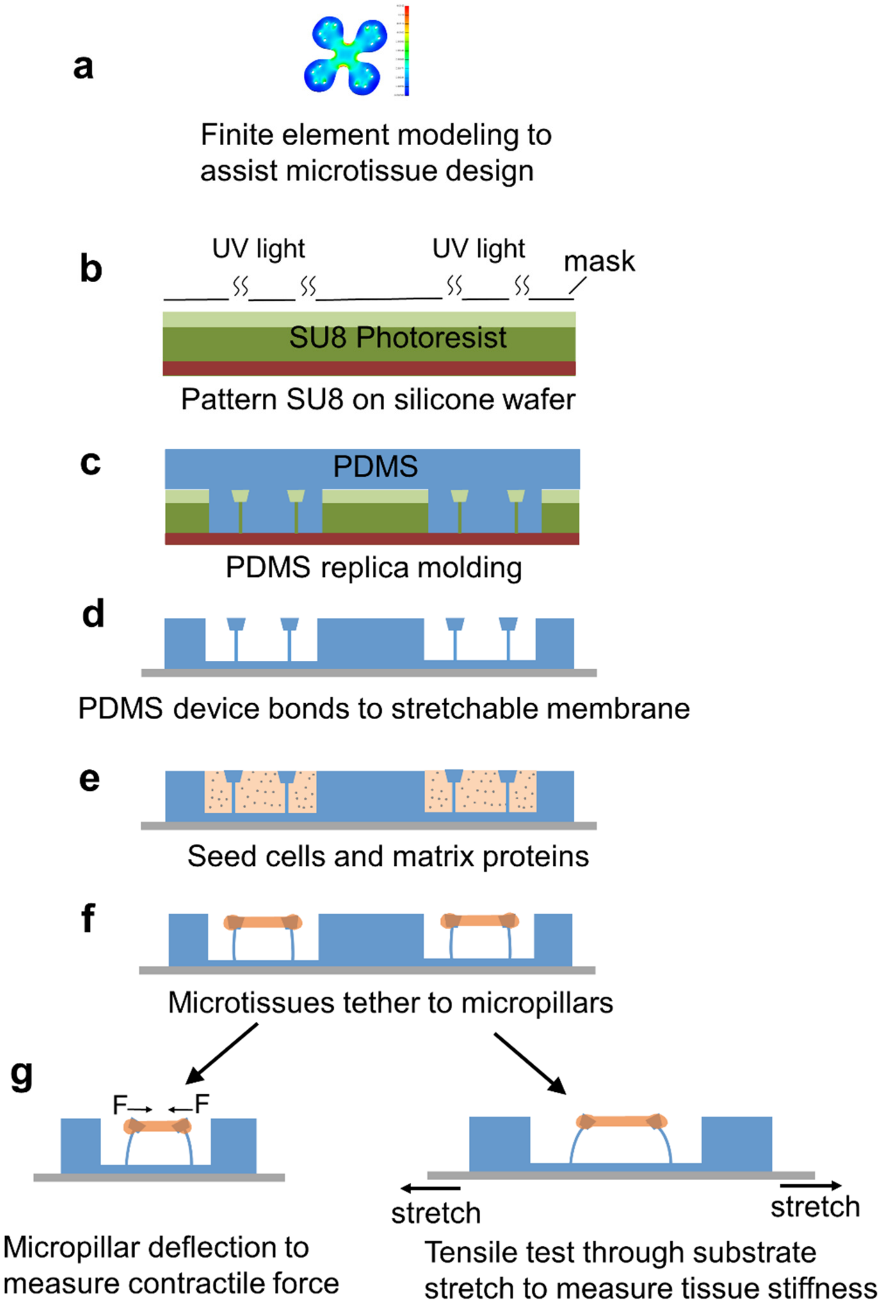

Supplementary Figure 3. Flow chart shows the steps of microtissue device design, fabrication and application in tissue mechanical property testing.

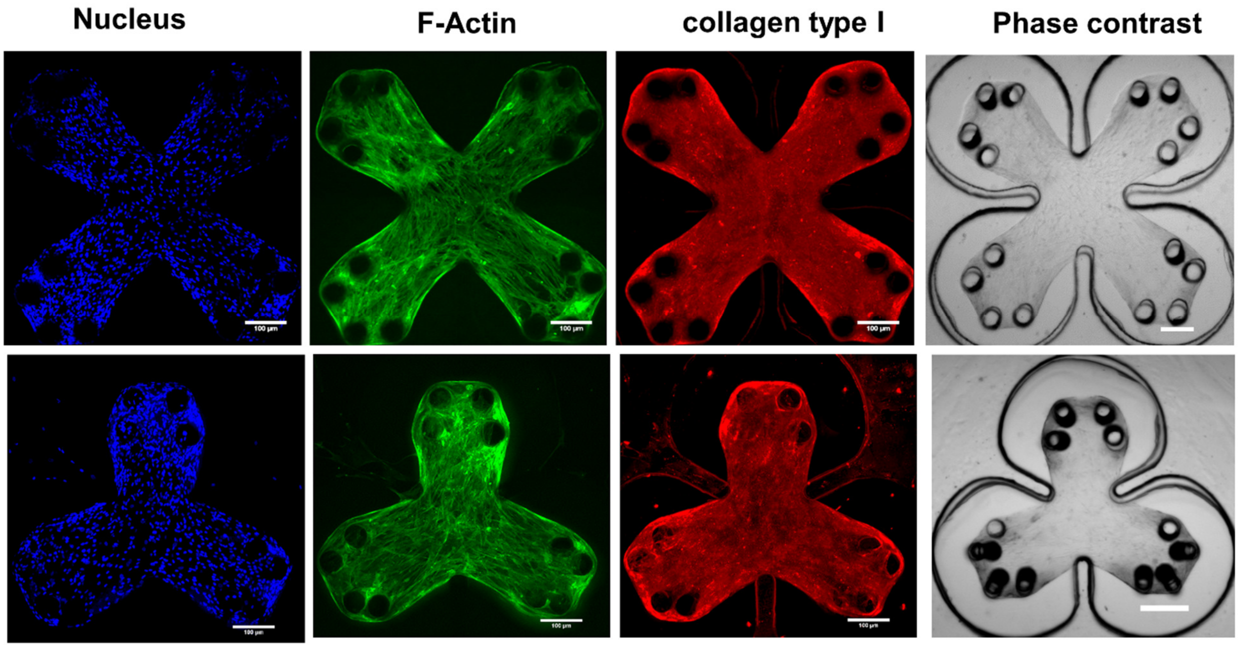

Supplementary Figure 4. Representative fluorescent confocal images and phase contrast images of a three leaflet microtissue (top row) and a four leaflet microtissue (bottom row). (Scale bar, 100μm).

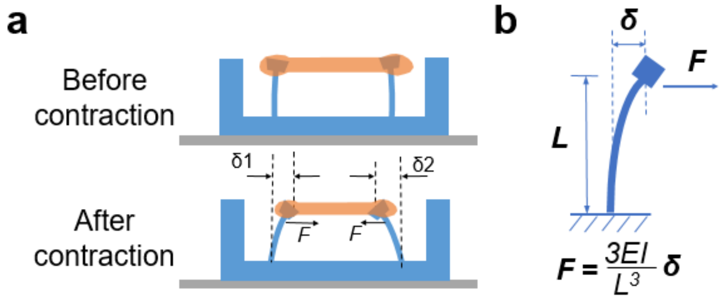

Supplementary Figure 5. Micropillar deflection (δ) is used to calculate microtissue contractile force according to cantilever bending theory. (**a**) Sideview of the microtissue and micropillars shows the measurement of micropillar deflection (δ). (**b**) Schematic shows the cantilever bending theory.

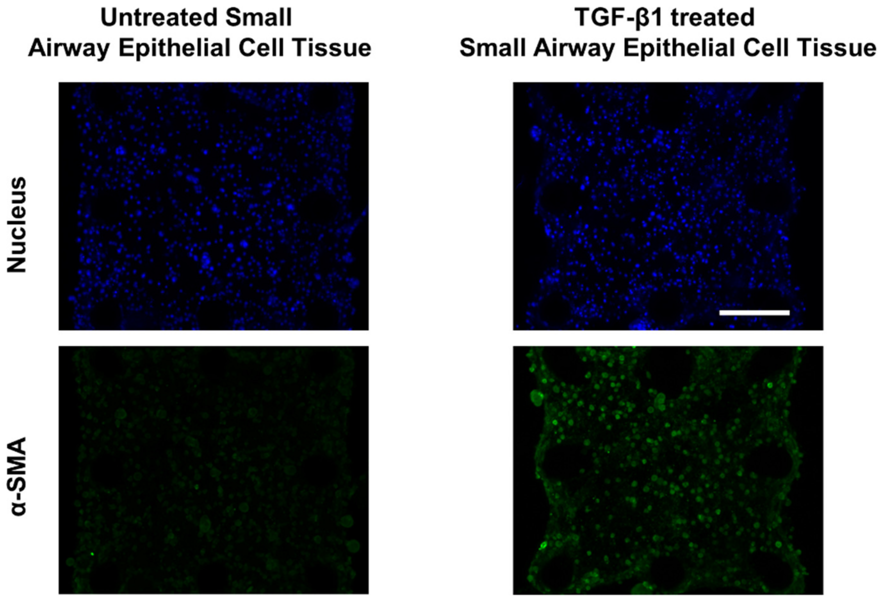

Supplementary Figure 6. The expression of fibrosis biomarker α-SMA in human lung small airway epithelial cell (SAECs)-populated collagen microtissues. There is no significant increase in α-SMA expression in TGF-β1 treated samples. (Scale bar, 200μm).

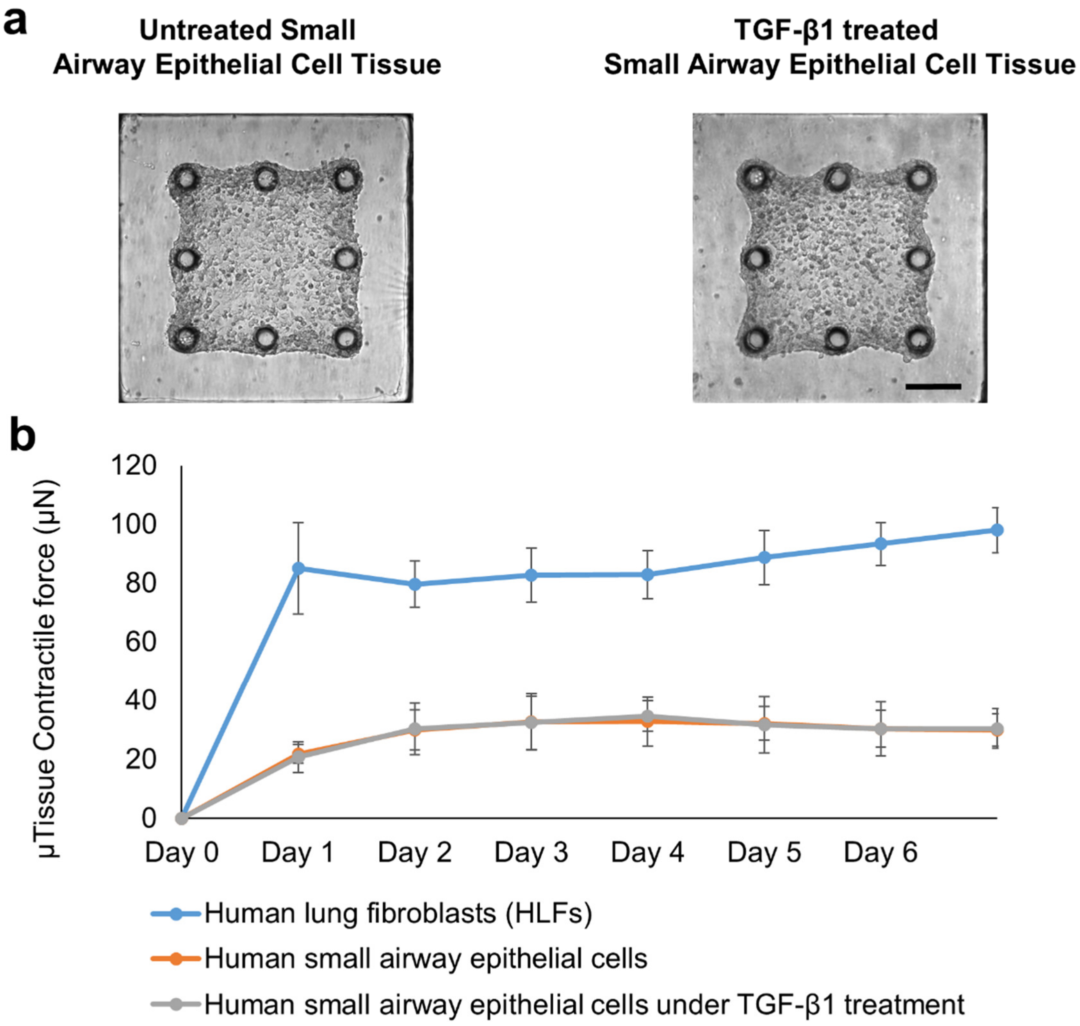

Supplementary Figure 7. Contractile force measurement of human lung small airway epithelial cell (SAECs)-populated collagen microtissues. (**a**) Phase contrast images of SAECs-populated microtissues under untreated and TGF-β1 treated conditions. (**b**) Time-lapsed contractile force measurement of SAECs-populated microtissues. The contractile force generated by SAECs-populated microtissues is low and around 20 – 30 μN as compared to the high contractile force of 90 – 100 μN generated by untreated human lung fibroblast-populated microtissue. Note the morphology of the SAECs remained cuboidal in the microtissue, unlike the spread morphology of the lung fibroblasts. (Scale bar, 200μm).

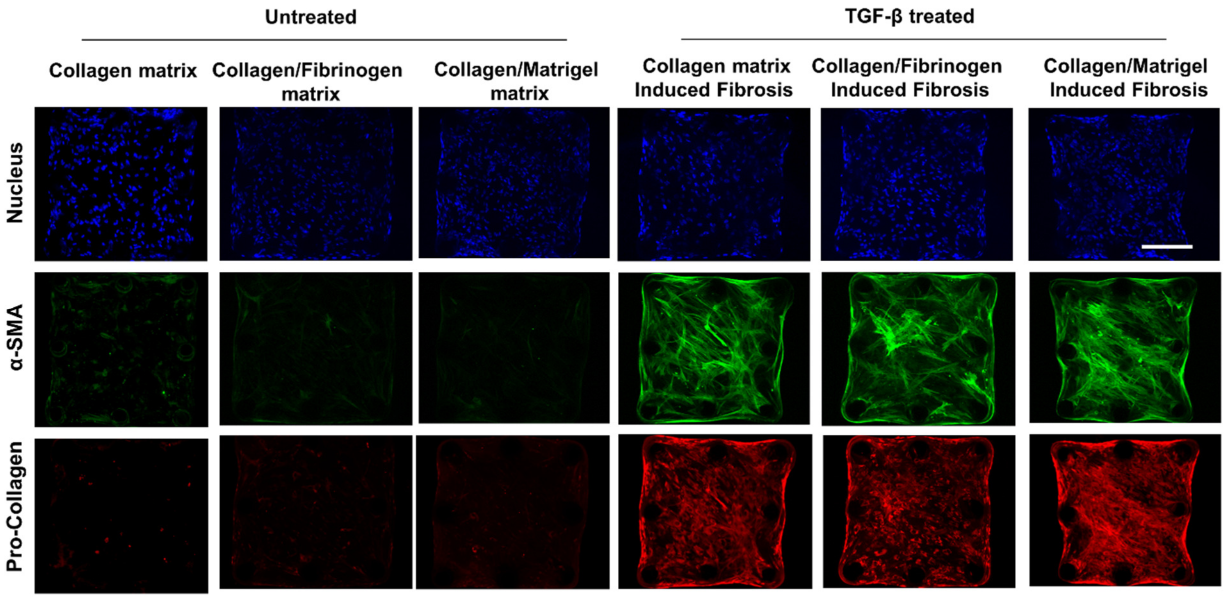

Supplementary Figure 8. The expressions of fibrosis biomarkers, α-SMA and pro-collagen, in lung fibroblast-populated microtissues fabricated using different ECM proteins. In untreated condition, biomarker expression is negative for all ECM formulas. Continuous TGF-β1 treatment induced strong expressions of α-SMA stress fibers and cytosolic pro-collagen in all three ECM formulas. (Scale bar, 200μm).

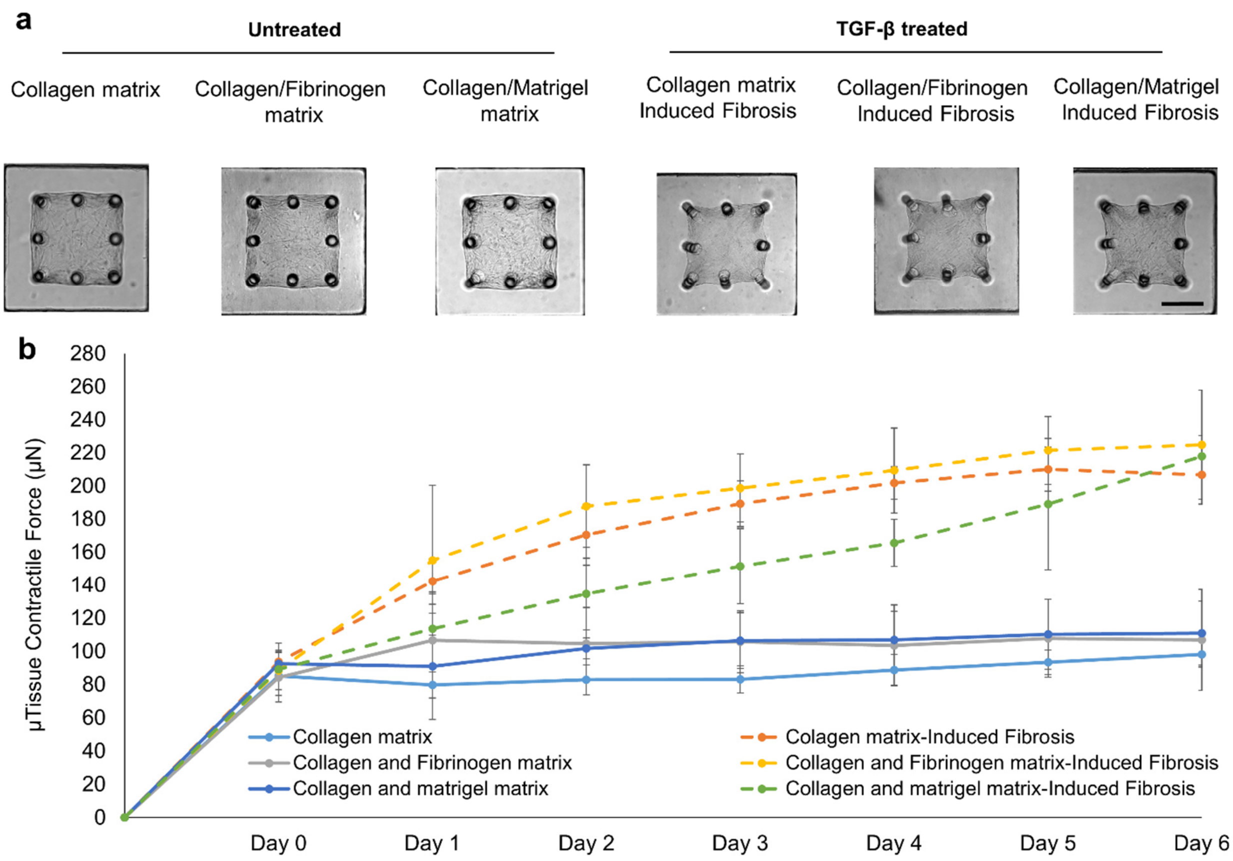

Supplementary Figure 9. Contractile force measurement of lung fibroblast-populated microtissues fabricated using different ECM proteins. (**a**) Phase contrast images of the microtissues. Note the obvious micropillar deflection in TGF-β1 treated condition for all three ECM formulas. (**b**) Time-lapsed contractile force measurement for the microtissues. In untreated condition, contractile forces are low and around 80 – 100 µN for all three ECM formulas. Continuous TGF-β1 treatment caused the contractile forces to increase to around 200 µN by day 6 for all three ECM formulas. (Scale bar, 200µm).

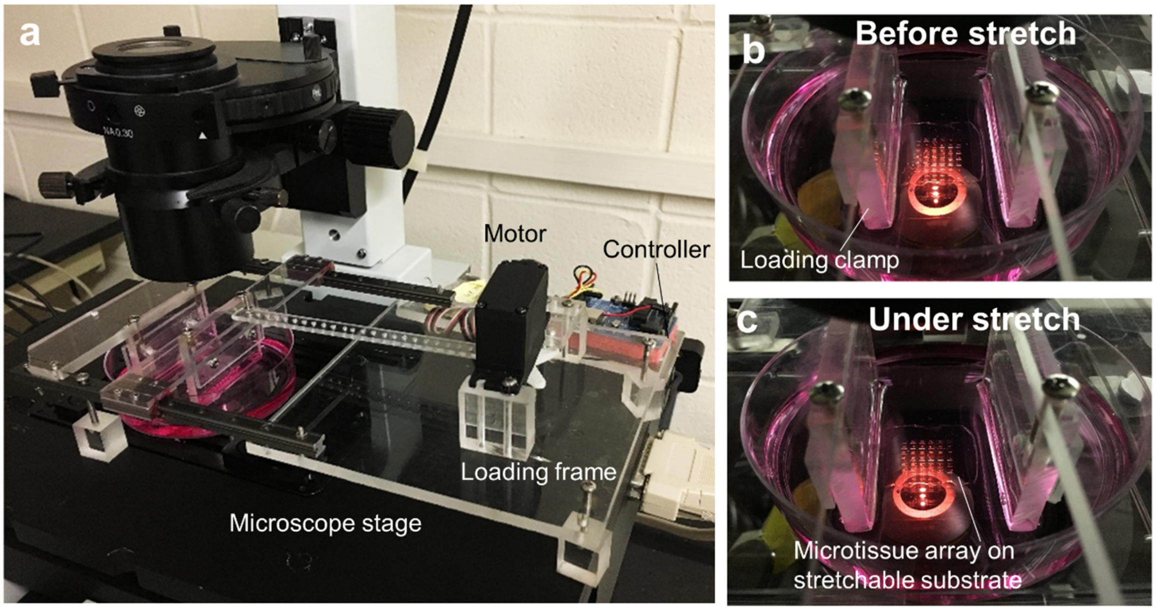

Supplementary Figure 10. Microtissue mechanical stretching system setup. (**a**) Custom-made stretching system was mounted on the microscope stage. A transparent stretchable silicone substrate was mounted on the loading frame driven by a DC motor controlled by an Arduino microcontroller. The mounting fixture allows the silicone substrate to be lowered to close to the bottom of a P100 petri-dish containing culture media. (**b**) Microtissue array before stretch. Microtissue array is directly above the objective to allow imaging. (**c**) Microtissue array under stretch.

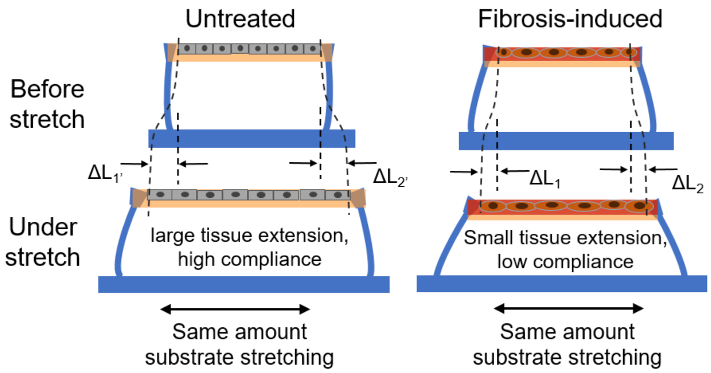

Supplementary Figure 11. Schematic shows mechanical stretching of membranous microtissue and the principle of tissue compliance measurement.

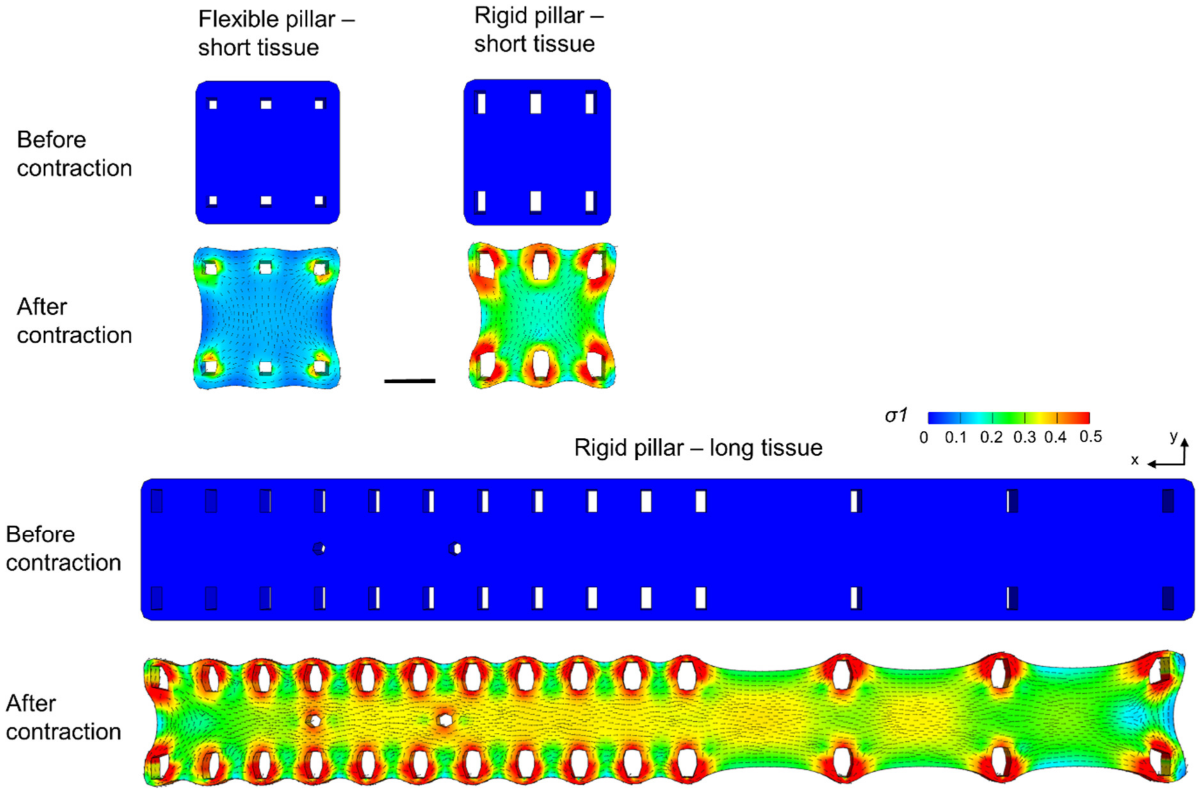

Supplementary Figure 12. Undeformed geometry and deformed geometry overlaid with FE simulated 1st principal stress distribution of a square microtissue supported by flexible micropillars, a square microtissue supported by rigid micropillars and a long microtissue supported by rigid micropillars. Scale bar is 500 μm.

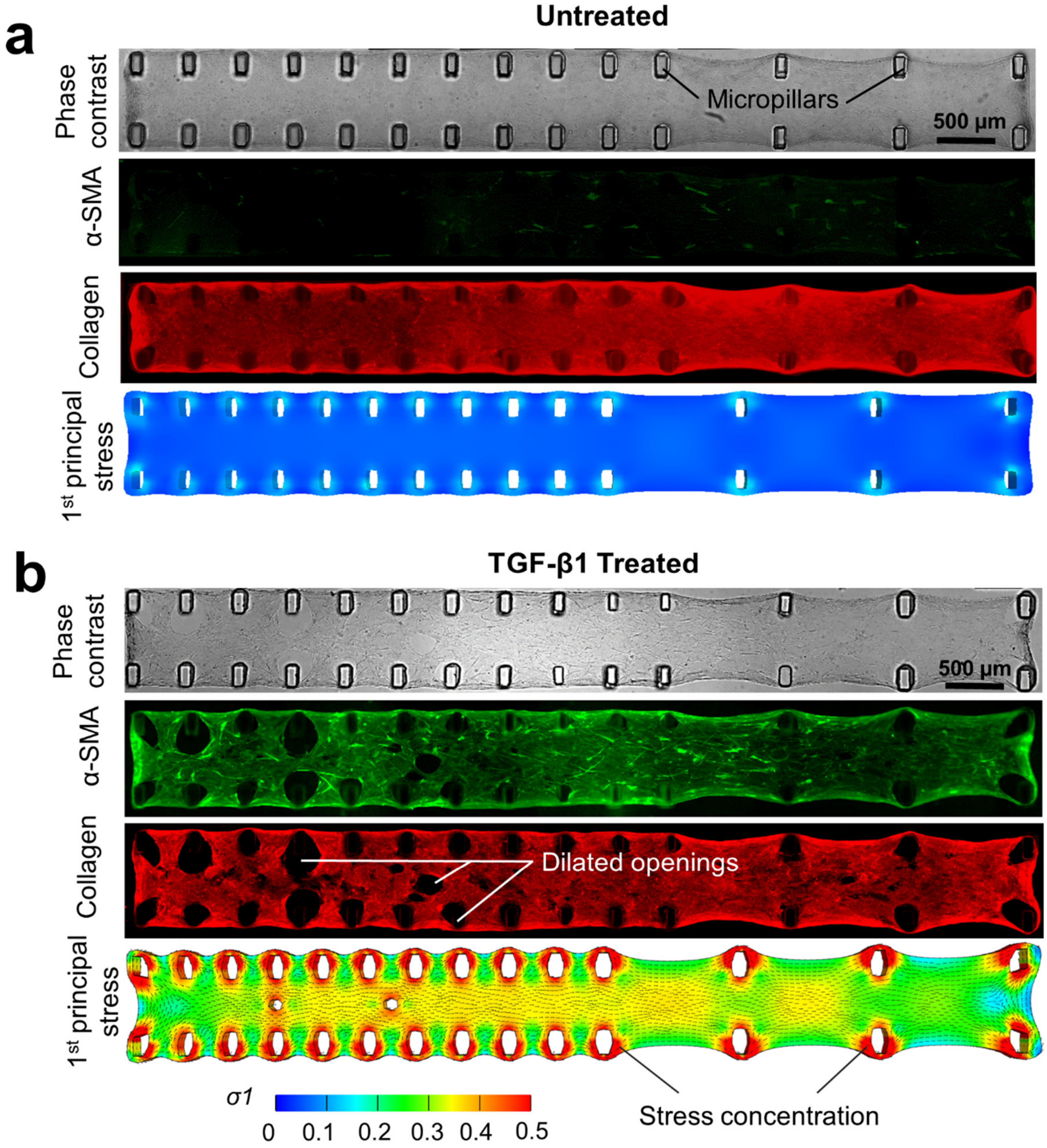

Supplementary Figure 13. (**a**) Phase contrast image, immunofluorescence images of α-SMA and collagen type-I and FE simulated 1st principal stress distribution of an untreated long microtissue. No apparent opening dilation can be observed around the micropillars. (**b**) Phase contrast image, immunofluorescence images of α-SMA and collagen type-I and FE simulated 1st principal stress distribution of a TGF-β1 treated long microtissue. Apparent opening dilation and high stress concentration can be observed consistently around the micropillars. Scale bar is 500 μm.

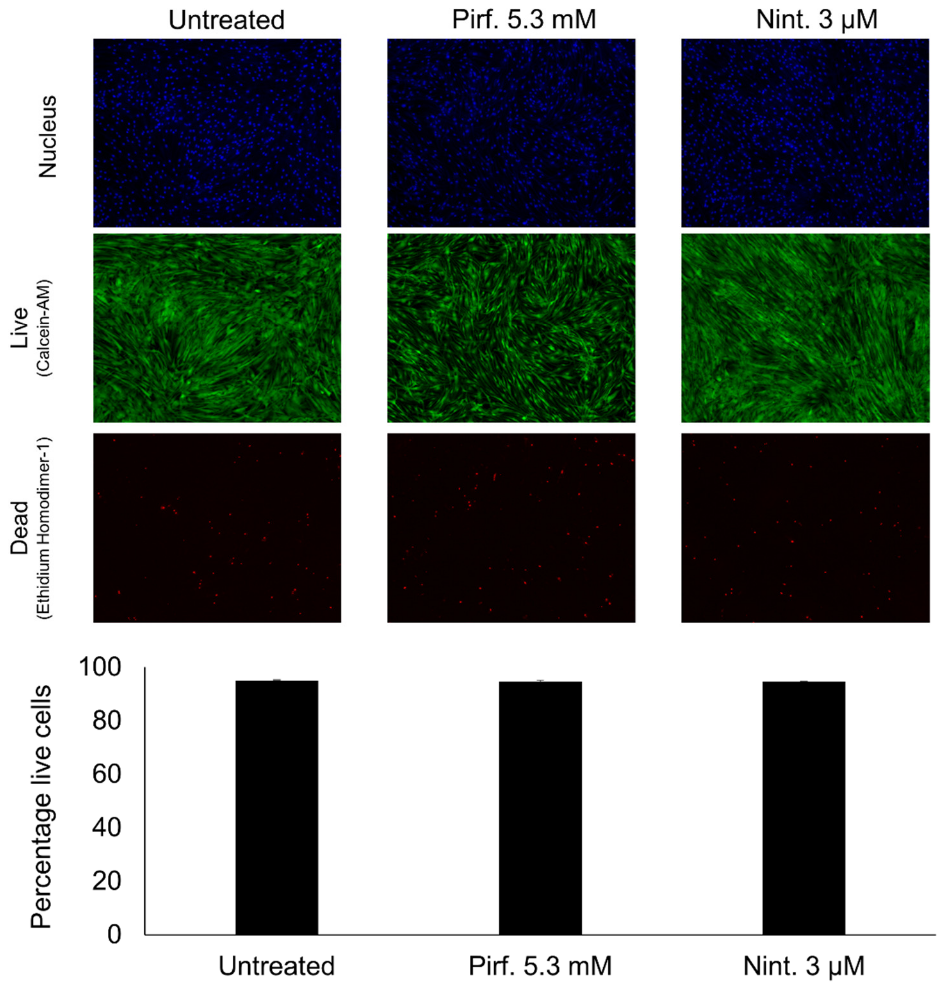

Supplementary Figure 14. Live/Dead assay of 2D culture of lung fibroblasts under different anti-fibrosis drug treatments. High cell viability remained after 6 days culture with anti-fibrosis drugs.

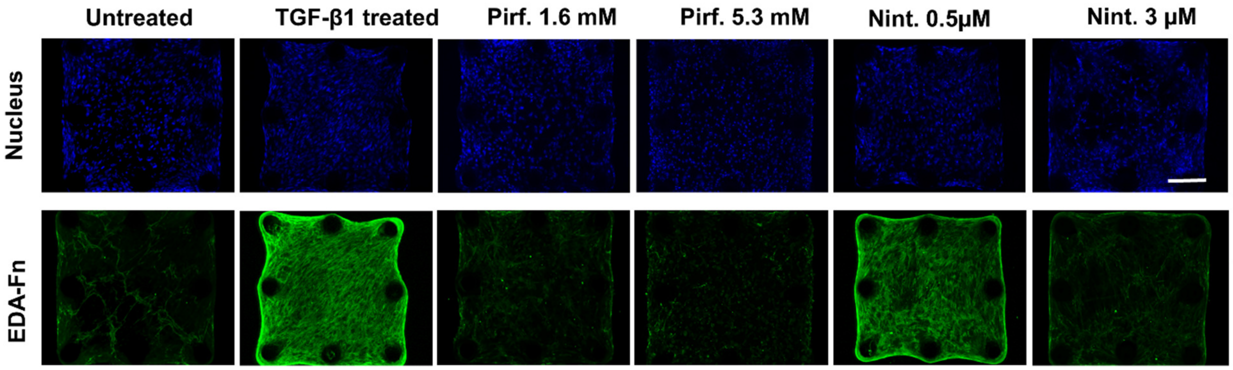

Supplementary Figure 15. Representative immunofluorescence images of nuclei and EDA-Fibronectin (Fn) of microtissues at day 6, with or without preventative anti-fibrosis treatments. (Scale bar, 200 μm).

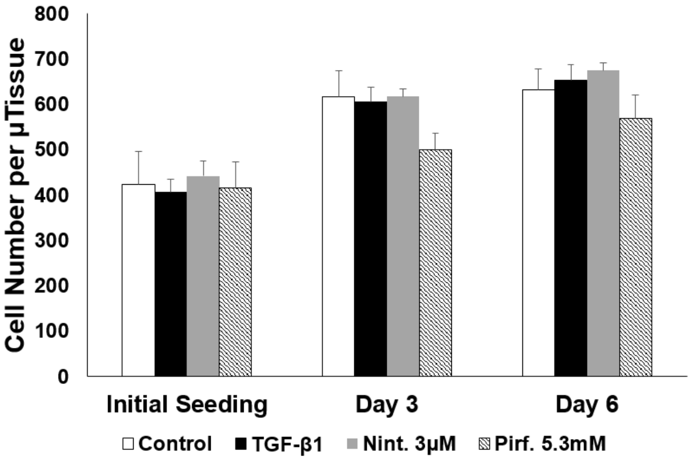

Cell Number	Initial Seeding	Day 3	Day 6
Control	423	617	633
TGF-β1	406	606	654
Nint. 3µM	443	618	675
Pirf. 5.3mM	417	500	570

Supplementary Figure 16. Measurement of cell proliferation under TGF-β1 and anti-fibrosis treatments over a 6 day culture period.

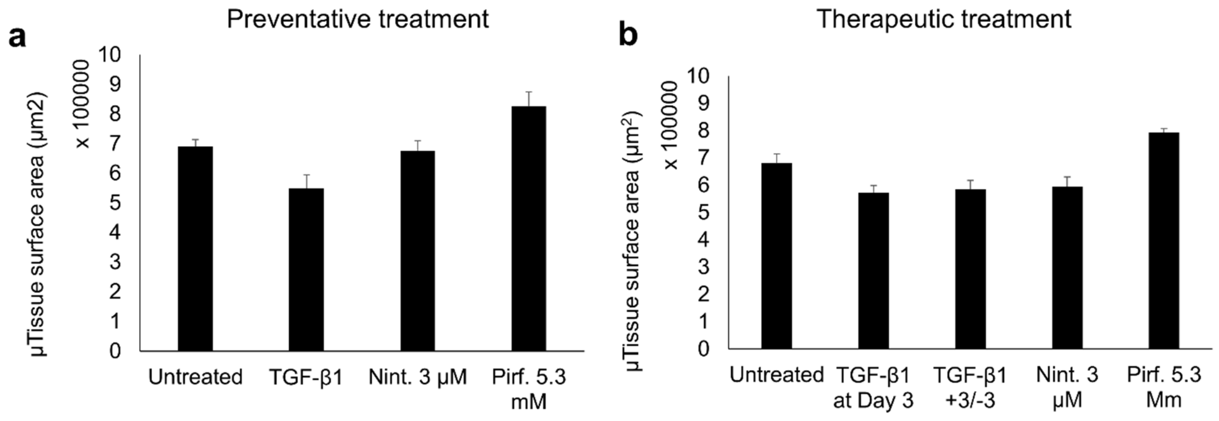

Supplementary Figure 17. Microtissue area as a measurement of the ECM compaction level. (**a**) Measured area for untreated and TGF-β1 treated microtissues and microtissues under preventative anti-fibrosis drug treatments. Anti-fibrosis drug treatments inhibited matrix compaction, as shown by the large microtissue area under Pirfenidone and Nintedanib treatment. (**b**) Measured area for untreated and TGF-β1 treated microtissues and microtissues under therapeutic anti-fibrosis drug treatments. Pirfenidone 5.3 mM treatment inhibited matrix compaction.

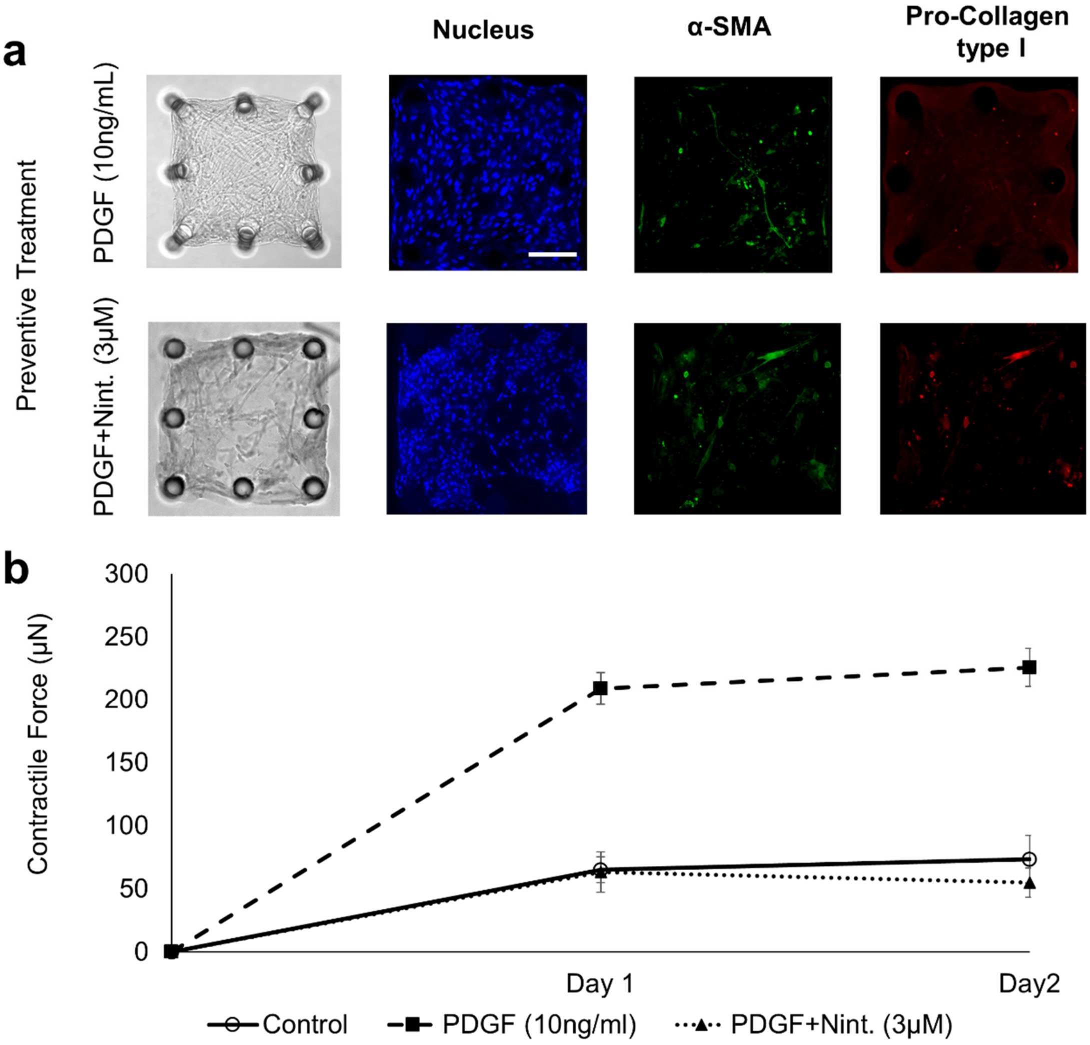

Supplementary Figure 18. (**a**) Representative fluorescent confocal images and phase contrast images of microtissues under PDGF or PDGF + Nintedanib treatment. (**b**) Microtissue contractile forces measured under untreated, PDGF treated or PDGF + Nintedanib treated conditions. Nintedanib strongly inhibited PDGF induced tissue contractility. Scale bar is 200 μm.

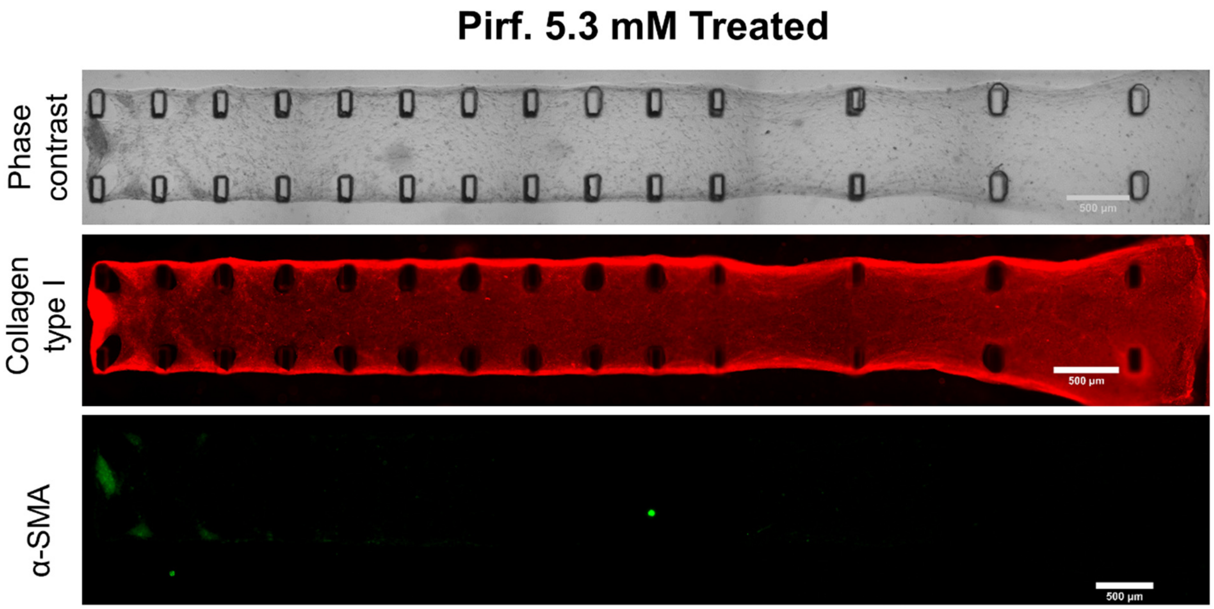

Supplementary Figure 19. Phase contrast image, immunofluorescence images of α-SMA and collagen type-I of a TGF-β1 induced long microtissue under Pirfenidone treatment. Pirfenidone treatment inhibited the fibrotic opening dilation. Scale bar is 500 µm.

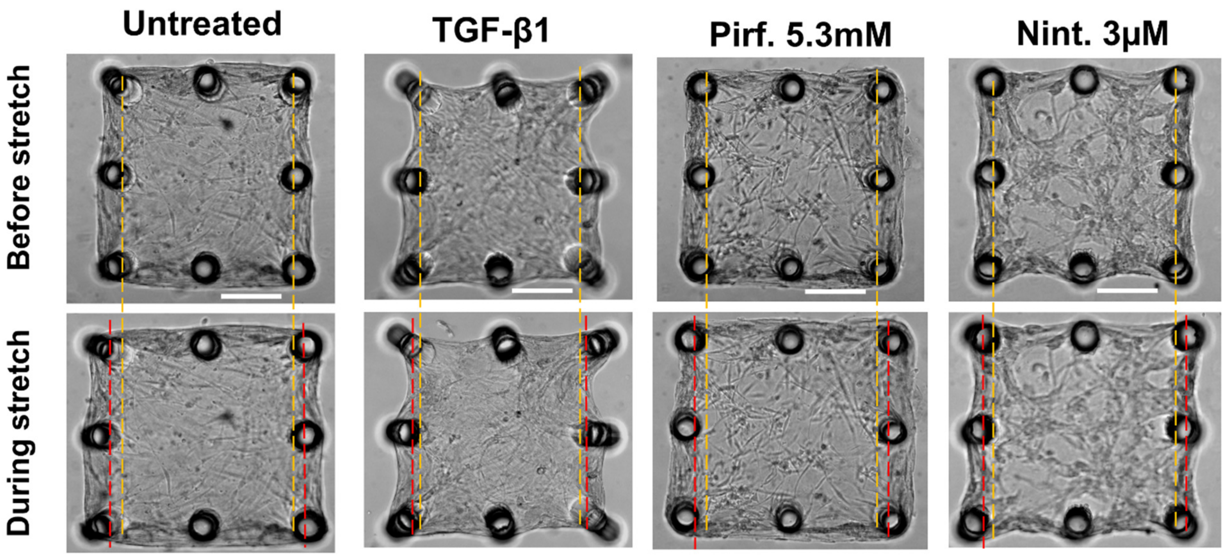

Supplementary Figure 20. Comparison of tissue compliance between microtissues under fibrosis induction and anti-fibrosis treatments. Phase contrast images show microtissues treated with TGF-β1 or different drugs before and during stretching. Stretching-induced extension (gaps between yellow and red lines for each microtissue) of Pirfenidone and Nintedanib treated microtissues was larger than that of TGF-β1 treated microtissue and similar to that of untreated sample, indicating restore of the compliance under anti-fibrosis treatments. (Scale bar, 200 μm).

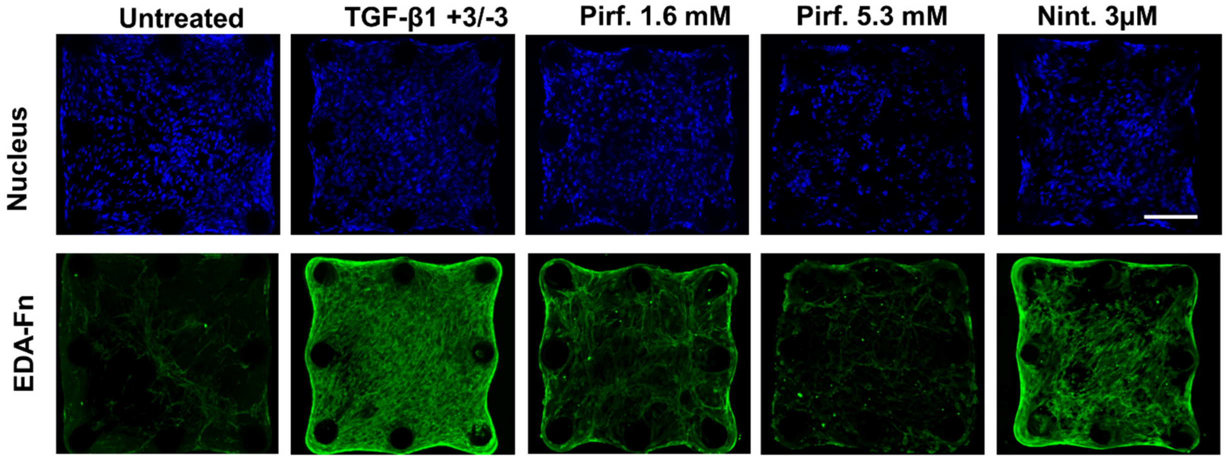

Supplementary Figure 21. Representative immunofluorescence images of nuclei and EDA-Fibronectin (Fn) of microtissues at day 6, with or without therapeutic anti-fibrosis treatments. (Scale bar, 200 μm).

Supplementary Note 1

Estimation of drug absoption in the PDMS

In the paper by Domansky et al. [1], PBS solution containing drugs was made in contact with the PDMS specimen at 1: 1 volume ratio for 3-days, and it was found that 33% of Pirfenidone was recovered from the PBS solution. In our microtissue system, the diameter of the PDMS device chamber, which contains the microtissue array and holds drug-containing culture media, is 2.5 cm. According to the above paper, the penetration depth of drugs into the PDMS is about 0.5 mm after 3 days of absorption. Hence, the PDMS volume that absorbs drugs is around 250 mm^3 in our system. However, the amount of culture media being added to the PDMS chamber is normally 2.5 mL = 2500 mm^3, which is 10 times the volume of the absorptive PDMS. The amount of Pirfenidone being absorbed by the PDMS should be (1-33%) x 10% = 6.6% of the total amount over a 3 days period. Since we refresh culture media every two days, it is expected that the actual Pirfenidone being absorbed by the PDMS should be less than 5%, which is negligible.

Supplementary references

1. Domansky, K. et al. SEBS elastomers for fabrication of microfluidic devices with reduced drug absorption by injection molding and extrusion. *Microfluid Nanofluidics* **21** (2017).
