# Supplementary material for: Fibrotic microtissue array to predict anti-fibrosis drug efficacy
Source: Nat Commun. 2018 May 25;9:2066. doi: 10.1038/s41467-018-04336-z (PMC5970268; doi:10.1038/s41467-018-04336-z)
Supplement: Supplementary file 3 — Description of Additional Supplementary Files [file 41467_2018_4336_MOESM3_ESM.pdf]

Description of Additional Supplementary Files

File Name: Supplementary Movie 1
Description: FE simulated thickness change of the four leaflet microtissue under cell-generated active contraction. Under active contraction, the tissue compacted freely through its thickness (Z axis), as demonstrated by the change in the z position of the two surfaces from 0 – 80 µm (thickness 80 µm) to 21.6 – 88.7 µm (thickness 67.1 µm). Scale bar shows the range of the Z position.

File Name: Supplementary Movie 2
Description: FE simulated geometry and stress evolution of the four leaflet microtissue under cell-generated active contraction. Under active contraction, tissue compaction in X-Y plane was restricted by micropillar-defined boundary conditions, leading to stress concentration. This was demonstrated by the appearance of large areas of high stresses (5 x 10^-3 A.U.) around the micropillars. Scale bar shows the range of the 1st principal stress.

File Name: Supplementary Movie 3
Description: Time-lapsed movie showing the formation process of a square microtissue. Cells started to spread and compact the ECM at 1-2 hours, leading to the formation of a shape-defined, membranous microtissue within 14 hours.
